# Isolated Thrombosis of the Superior Mesenteric Vein

**DOI:** 10.7759/cureus.7477

**Published:** 2020-03-30

**Authors:** Rajesh Essrani, Shehriyar Mehershahi, Rajesh Essrani, Anuraj M Sudhakaran, Asif Mehmood

**Affiliations:** 1 Internal Medicine, Geisinger Medical Center, Danville, USA; 2 Internal Medicine, Lehigh Valley Health Network, Allentown, USA; 3 Gastroenterology, BronxCare Health System, Bronx, USA; 4 Internal Medicine, BronxCare Health System, Bronx, USA; 5 Internal Medicine, Abington Hospital - Jefferson Health, Abington, USA

**Keywords:** acute pancreatitis, superior mesenteric vein thrombosis, mesenteric venous thrombosis, abdominal pain

## Abstract

Most cases of pancreatitis are mild and self-limited. On the other hand, a few patients with pancreatitis may develop vascular complications. Splenic vein thrombosis is the most common vascular complication of acute pancreatitis. Isolated superior mesenteric vein thrombosis is rare and can lead to gut ischemia and necrosis if not timely diagnosed and managed. We report the case of a 40-year-old patient who presented to the hospital with abdominal pain due to acute pancreatitis, leading to superior mesenteric vein thrombosis, which was timely diagnosed and treated with a good outcome.

## Introduction

Acute pancreatitis is an acute inflammatory process of the pancreas. It can present as mild, moderate, or severe. Acute pancreatitis is usually mild in severity and patients recover in three to five days without complications or organ failure. However, 20% of patients have moderately severe or severe acute pancreatitis with local or systemic complications or organ failure. Mild cases are usually treated with conservative measures; severe cases may require admission to the intensive care unit, or even surgery, to deal with impending complications. Acute pancreatitis can result in many vascular problems, such as splanchnic vein thrombosis (splenic, portal, and/or superior mesenteric veins) and pseudoaneurysm [1-2]. Isolated thrombosis of the superior mesenteric vein (SMV) without involving the splenic or portal vein is very rare and is usually seen with intra-abdominal sepsis or pancreatic neoplasms [1]. It should never be missed due to the fatal complication of mesenteric ischemia and bowel infarction [1]. We report a case of acute pancreatitis leading to SMV.

## Case presentation

**Figure 1:**
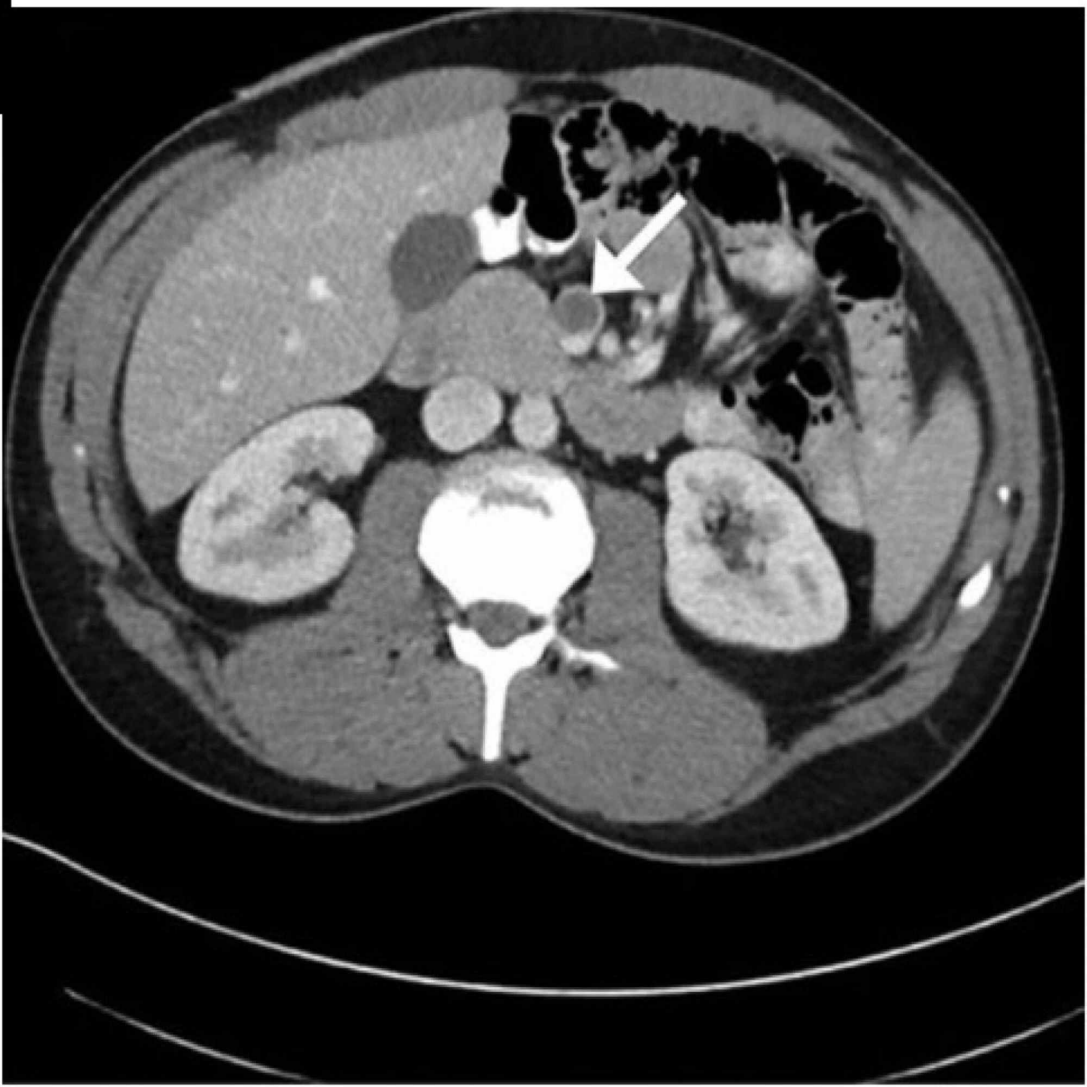
Computed tomography (CT) of the abdomen/pelvis showed pancreatitis with normal biliary ducts and an absence of opacification of the superior mesenteric vein (SMV)

A 40-year-old man with a past medical history of tobacco abuse was admitted with complaints of the sudden onset of epigastric pain with radiation to the back associated with nausea and vomiting of two days' duration. On examination, his blood pressure was 156/76 with a respiratory rate of 13, saturation 91% on room air, and a pulse of 101. The abdomen was soft but tender in the epigastric area with no right upper quadrant tenderness and bowel sounds decreased in all quadrants. Blood work showed a hemoglobin of 15.1 g/dL (normal: 14 - 17 g/dL), hematocrit 45.2% (normal: 41% - 51%), WBC - 17.04 K/uL (80% neutrophils) (normal: 3.3 - 8.7 K/uL), platelets - 600 K/uL (normal: 147 - 347 K/uL), serum aspartate aminotransferase (AST) - 24 U/L (normal: 0 - 35 U/L), alanine aminotransferase (ALT) - 22 U/L (normal: 0 - 35 U/L), alkaline phosphatase - 144 U/L (normal: 36 - 92 U/L), lactate - 1.2 mmol/L (normal: 0.5 - 1 mmol/L), triglyceride level - 86 mg/dL (normal: < 150 mg/dL), low-density lipoprotein (LDL) - 160 mg/dL (normal: ≤ 130 mg/dL), calcium - 9.1 mg/dL (normal: 9 - 10.5 mg/dL), blood urea nitrogen (BUN) - 30 mg/dL (normal: 8 - 20 mg/dL), creatinine - 1.3 mg/dL (normal: 0.7 - 1.3 mg/dL), sodium - 139 mmol/L (normal: (136 - 145 mmol/L), and lipase - 600 U/L (normal: less than 95 units/L). Computed tomography (CT) of the abdomen/pelvis showed pancreatitis with a normal biliary duct and an absence of opacification of the SMV (Figure 1). He denied any personal or family history of coagulopathy and denied alcohol intake. He was treated with pain medications and lactated Ringer's solution at 5 mL/kg per hour. Hematology recommended low-molecular-weight heparin, 1 mg/kg twice a day, and Coumadin. His condition improved in three days and he was discharged home with Coumadin to complete three months of treatment.

## Discussion

Mesenteric venous thrombosis is seen mainly in middle-aged patients presenting with vague symptoms, which makes this a hard diagnosis to make in the acute care setting. There are several risk factors which include hypercoagulable states, local trauma (e.g., splenectomy), local intra-abdominal inflammatory process (e.g., pancreatitis), and recent surgery (causing stagnant blood flow) [3]. Our patient had acute pancreatitis causing SMV thrombosis.

SMV is very uncommon with an incidence of 5% - 15% of all cases of mesenteric vessel occlusive disease [4]. It is a fatal condition that leads to life-threatening complications if not diagnosed or treated in a timely manner. It is caused by various mechanisms, such as venous compression, and an imbalance between fibrinolysis and coagulation because of local inflammatory processes [2, 5-6].

The SMV thrombosis diagnosis is difficult due to the lack of specificity of clinical symptoms. It usually presents as nausea, vomiting, and vague abdominal pain that can easily be masked by pancreatitis, as in our patient. The hallmark is pain out of proportion to physical findings [7].

Magnetic resonance (MR) venography is the most accurate imaging study for the diagnosis of mesenteric venous thrombosis but contrast-enhanced CT of the abdomen is used as an initial screening study due to a sensitivity of more than 90% [3]. The CT scan can detect focal or segmental bowel wall ischemia, thus assisting in the decision-making of operative and nonoperative approaches [8].

SMV thrombosis can present acutely, subacutely, or more chronically. The treatment of SMV thrombosis (acute and subacute) is predominantly conservative, consisting of systemic anticoagulation, bowel rest, and serial observation for any signs of clinical deterioration. Surgical exploration is limited to those patients with definite signs of bowel infarction [2, 5].

## Conclusions

Acute pancreatitis may be complicated by venous thrombosis. The isolated superior mesenteric vein is very rare and emergent. It can be diagnosed by a CT scan of the abdomen. Anticoagulant therapy is useful if the diagnosis is made before the stage of bowel infarction and lowers mortality. The physician should be vigilant about complications of pancreatitis in every patient.
